# Seeds as space‐time travelers: How does evolution balance the joint benefits and trade‐offs of dormancy and dispersal?

**DOI:** 10.1002/ajb2.70175

**Published:** 2026-03-11

**Authors:** Sofia Backlund, Sean Stankowski, Rosina Soler

**Affiliations:** ^1^ Institute of Science and Technology Austria Am Campus 1 Klosterneuburg 3400 Austria; ^2^ Department of Genetics, Evolution and Environment University College London WC1E 6BT

**Keywords:** life‐history strategies, population dynamics, seed banks, trade‐offs

Seeds face a fundamental challenge: once released, they enter environments that are highly variable in both space and time. Their ability to establish depends on escaping stressful conditions and encountering environments that are suitable for growth. Two strategies allow seeds to navigate unpredictability (Figure 1). Dispersal moves them through space via wind, animals, water, or other vectors, whereas dormancy—defined here as the delay of germination through the programmed shutdown of embryonic metabolism and growth—allows seeds to disperse through time.

**Figure 1:**
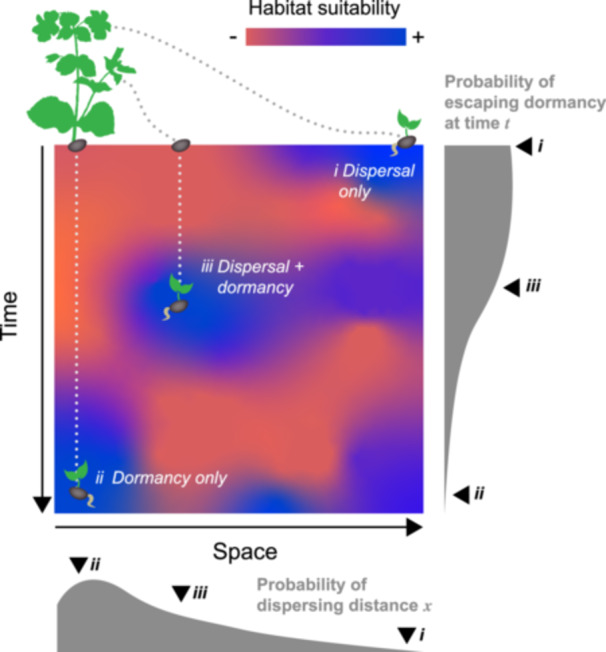
The movement of seeds in space‐time. The hypothetical two‐dimensional space shows how habitat suitability varies across space and time. Seeds can avoid unsuitable conditions in the present by (*i*) dispersing to a more suitable location or (*ii*) remaining dormant until conditions change. However, a mix of both processes (*iii*) can also give individuals a great chance of reaching a suitable habitat. Gray‐shaded areas represent hypothetical distributions showing the probability of dispersing to distance *x* and of escaping dormancy at time *t*. In our example, the probability of observing outcome *iii* is greater than observing outcomes *i* or *ii*.

Although often treated as distinct processes, dispersal and dormancy are deeply intertwined. Theory shows that both strategies can act jointly to buffer populations against environmental variability and local extinctions (Buoro and Carlson, 2014). Dispersal and dormancy can also shape gene flow and population dynamics (Saastamoinen et al., 2018), yet their combined effects remain poorly understood. Dormancy can reduce kin competition and promote altruistic behavior (Twyman and Gardner, 2023), echoing classic insights on spatial dispersal (Hamilton and May, 1977).

However, much remains to be learned about how dormancy and dispersal allow seeds to navigate environmental heterogeneity through space and time, whether these strategies can be optimized by selection, and when a joint strategy, involving a combination of dormancy and dispersal, might be optimal. Here, we highlight a few open questions that we see as the most important.

## TO WHAT EXTENT ARE DORMANCY AND DISPERSAL GENETICALLY DETERMINED?

Evolutionary models assume that dormancy and dispersal are heritable, yet empirical support for this is uneven. Studies show substantial genetic variation in the depth of dormancy (Clerkx et al., 2004), yet most evidence comes from model or cultivated species studied in controlled environments. This highlights the need for more estimates from natural populations. By contrast, the heritability of seed dispersal is poorly understood. Dispersal is more difficult to measure and likely has a complex genetic basis (Saastamoinen et al., 2018). A few experimental studies, mainly in wind‐ or animal‐dispersed species, show genetic variation in dispersal‐related traits, but direct heritability estimates remain rare (Johnson et al., 2019).

A clearer picture of the genetic architecture of dormancy and dispersal is also needed to predict their joint responses to selection (Conner, 2002). If dormancy and dispersal are underpinned by largely independent genetic architectures, then it is possible for selection to optimize them independently. However, if they have a shared genetic architecture, then underlying genetic correlations will cause selection on one trait to generate an indirect evolutionary response in the other, potentially constraining adaptation (Conner, 2002). Spatially explicit pedigree studies designed to infer parent‐offspring relationships in the wild would help reveal whether dispersal and dormancy can evolve independently or whether their evolution is limited by a shared genetic basis (Bradburd and Ralph, 2019).

## WHAT DRIVES TRADE‐OFFS BETWEEN DISPERSAL AND DORMANCY?

Seed traits may also reflect a fundamental trade‐off between moving through space and persisting through time. For example, within a given species, one might expect that (1) lighter seeds are more likely to disperse long distances and (2) heavier seeds are better buffered for persistence through long bouts of dormancy because they contain greater nutrient reserves (Thomson et al., 2011; Chen et al., 2020). On the other hand, smaller seeds can exhibit deeper dormancy in some species (Krzyszton et al., 2024), highlighting that the direction of trade‐offs is not always intuitive.

Currently, little is known about how most functional traits—such as appendages, buoyancy, seed‐coat thickness, or physiological mechanisms like water uptake, hormonal regulation, and nutrient storage—shape this balance. If traits that enhance persistence can compensate for limited spatial movement, then species with weak dispersal might evolve stronger dormancy or other longevity‐enhancing traits. Understanding how these traits interact is key to revealing how plants navigate the joint challenges of dispersal in space and time.

## HOW DO ENVIRONMENTAL CUES INTERACT TO SHAPE DORMANCY?

Environmental cues that influence dormancy and dispersal have been studied extensively under controlled conditions, but natural environments present far more complex and interacting signals. Understanding how seeds integrate multiple cues under realistic conditions remains a major challenge.

Dormancy is generally viewed as an adaptation to environmental variability, yet the selective pressures that shape dormancy strategies are still unclear. Predictable environments (either harsh or favorable) may favor plastic responses to cues such as temperature or photoperiod, whereas unpredictable conditions may select for bet‐hedging, spreading germination through time (Evans and Dennehy, 2005). Because real environments combine predictable and unpredictable elements, plasticity and bet‐hedging should interact, but their joint evolution has rarely been examined.

Seeds also integrate environmental information both within and across generations through maternal effects, which can alter traits such as size and dormancy in response to the mother plant's environment (Chen et al., 2025). However, we still know little about how such maternal influences interact with spatial dispersal or with fine‐scale environmental variability in nature.

## HOW DO ENVIRONMENTS VARY AT DIFFERENT SPATIOTEMPORAL SCALES?

A major challenge in understanding seed responses to the environment is the mismatch between the coarse scales of environmental data collection and the fine scales at which seeds perceive their environment. Macroscale variables describe broad climatic patterns, and mesoscale factors capture local heterogeneity, but it is the microenvironment experienced by individual seeds that forms the true selective arena. Understanding dormancy and dispersal, therefore, requires integrating information across these nested spatial and temporal scales.

Species also differ markedly in how they experience environmental heterogeneity. The seeds of a wind‐dispersed species will encounter a broad mosaic of microsites, whereas a gravity‐dispersed seed may encounter only the highly localized environment near its parent. This creates a tension between species‐specific environmental perception and the assumptions used in models and experiments, which often treat all seeds as sensing their surroundings in the same way (Long et al., 2015; Twyman and Gardner, 2023). In reality, risk and heterogeneity are species‐relative, shaping how different plants evolve dormancy and dispersal as complementary strategies for survival.

## WHEN ARE SPACE AND TIME CONTINUOUS?

Most theoretical population models are nonspatial, using well‐mixed or patch‐based populations (Bradburd and Ralph, 2019). Yet real populations occupy continuous landscapes, so dividing them into discrete patches is often arbitrary and biologically unrealistic. Capturing trade‐offs between dormancy and dispersal, therefore, requires models that operate in continuous space.

By contrast, representing time as discrete often makes biological sense for plants, because seasonal boundaries (e.g., winter dormancy) naturally structure the life cycle. However, within‐season processes such as germination timing or seed production may require continuous‐time approaches, especially when these dynamics directly affect dispersal in space and time.

Empirical meta‐analyses often treat dispersal and dormancy as binary traits (e.g., long vs. short dispersal and dormant vs. nondormant; Chen et al., 2020), yet both vary continuously within species and can occur across multiple steps, such as secondary dispersal or dormancy cycling. Understanding how dormancy and dispersal interact and trade off thus requires analyzing their full distributions rather than simplifying them to presence‐absence categories.

## WHAT ARE THE EVOLUTIONARY INTERACTIONS BETWEEN MATING SYSTEMS, SEX ALLOCATION, DISPERSAL, AND DORMANCY?

Mating systems, sex allocation, dispersal, and dormancy all influence inbreeding, yet their interactions remain largely unexplored. For example, many plants avoid inbreeding through self‐incompatibility systems, but long‐distance dispersal also reduces mating among relatives (Cartwright, 2009). Thus, species with long‐range dispersal may lose self‐incompatibility, whereas self‐incompatible species are less likely to be negatively impacted by inbreeding costs under limited dispersal. Similar interactions may occur with dormancy, but this has not been tested.

Inbreeding costs can also favor the evolution of dioecy. Spatial dispersal is thought to play a role here as well, as models show that both seed and pollen must disperse widely for dioecy to evolve. By contrast, dispersal limitation favors cosexuality (Fromhage and Kokko, 2010). Whether temporal dispersal can substitute for spatial dispersal in this context remains an open question.

Biased sex ratios in dioecious species, sometimes detectable even at the seed stage, suggest that early developmental or ecological processes, including dispersal and dormancy, may shape sex allocation. Understanding how mating systems, sex allocation, and dispersal and dormancy interact could reveal new evolutionary dynamics, ripe for both theoretical and comparative study.

## HOW DOES POLLEN DISPERSAL INTERACT WITH SEED DISPERSAL AND DORMANCY?

Pollen dispersal is largely overlooked when comparing dispersal in space and time. Existing models typically assume asexual reproduction and treat dispersal as a single step, even though gene flow in plants occurs first via pollen and only later via seeds. This raises an important question: To what extent can pollen movement provide some of the same benefits as seed dispersal, thereby reducing selection for long‐distance seed movement or extensive dormancy?

Understanding optimal seed dispersal strategies, therefore, requires recognizing that pollen and seed dispersal operate independently and often at very different spatial scales. New theoretical models should explicitly incorporate both steps, and empirical syntheses should account for pollen movement when comparing spatial and temporal dispersal. Including pollen dispersal is especially important for predicting not just population persistence, but also patterns of genetic diversity.

## CONCLUSIONS

Addressing these questions requires treating dispersal and dormancy together. Progress will come from integrating theory, physiology, ecology, and population genomics across spatial and temporal scales. This will clarify how plants use the two strategies, both individually and in combination, to cope with environmental uncertainty and improve predictions of their responses to change. The insights gained from plants will offer a powerful framework for studying the evolution of dormancy‐dispersal dynamics across the tree of life.

## AUTHOR CONTRIBUTIONS

S.B. and R.S. conceptualized the manuscript. S.S. developed the figure. All authors further developed the idea and contributed to writing the essay.

## Data Availability

No data are associated with this manuscript.
